# Sources of Discrepancy between Retinal Nerve Fiber Layer and Bruch’s Membrane Opening-Minimum Rim Width Thickness in Eyes with Glaucoma

**DOI:** 10.1016/j.xops.2024.100601

**Published:** 2024-08-22

**Authors:** Iris Zhuang, Maryam Ashrafkhorasani, Vahid Mohammadzadeh, Kouros Nouri-Mahdavi

**Affiliations:** Glaucoma Division, Stein Eye Institute, David Geffen School of Medicine, University of California at Los Angeles, Los Angeles, California

**Keywords:** Bruch’s membrane opening-minimum rim width, Glaucoma, Mismatch, OCT, Retinal nerve fiber layer

## Abstract

**Purpose:**

To compare the discrepancies between circumpapillary retinal nerve fiber layer (RNFL) and Bruch’s membrane opening-minimum rim width (BMO-MRW) thickness in glaucoma eyes.

**Design:**

A cross-sectional observational study.

**Subjects:**

One hundred eighty-six eyes (118 patients) with glaucoma.

**Methods:**

OCT optic nerve head volume scans of patients enrolled in the Advanced Glaucoma Progression Study at the final available visit were exported. The RNFL and BMO-MRW measurements were averaged into corresponding 7.5° sectors, and the nasal sector data were excluded from analyses. A 2-stage screening process was used to identify true mismatches between the RNFL and BMO-MRW measurements, in which either the RNFL or BMO-MRW value was in the less than first percentile range while its counterpart was in the greater than first percentile range on the temporal-superior-nasal-inferior-temporal curve. The prevalence of these mismatches was mapped, and corresponding images were reviewed to determine the underlying cause of these discrepancies.

**Main Outcome Measures:**

Proportion of mismatches between RNFL and BMO-MRW, location of mismatches between RNFL and BMO-MRW, anatomical causes of mismatches between RNFL and BMO-MRW.

**Results:**

Mismatch analysis revealed true mismatches between RNFL and BMO-MRW in 7.7% of sectors. High BMO-MRW with low corresponding RNFL mismatches were most frequently located at the 45° and 322.5° sectors, whereas high RNFL with corresponding low BMO-MRW mismatches peaked at the 75° sector. Large blood vessels accounted for 90.9% of high RNFL with low BMO-MRW mismatches. Small to large blood vessels accounted for 62.9% of high BMO-MRW with low RNFL mismatches; the remaining mismatches could be attributed to retinoschisis or inclusion of outer retinal layers in BMO-MRW measurements.

**Conclusions:**

Although overall agreement between RNFL and BMO-MRW measurements is good in areas with advanced damage, blood vessels and other anatomical factors can cause discrepancies between the 2 types of structural measurements and need to be considered when evaluating the utility of such measurements for detection of change.

**Financial Disclosure(s):**

Proprietary or commercial disclosure may be found in the Footnotes and Disclosures at the end of this article.

Glaucoma is a progressive optic neuropathy that manifests as degeneration of the retinal ganglion cells (RGCs) and their axons, known as the retinal nerve fiber layer (RNFL). This degeneration leads to thinning of the RNFL and the neuroretinal rim at the level of the optic nerve head (ONH). Such changes can be visualized and quantified with OCT. OCT has become an essential clinical tool for glaucoma management.

One of the most established and widely used OCT biomarkers for monitoring glaucomatous damage is circumpapillary RNFL thickness.^1^, ^2^, ^3^ The Bruch membrane opening-minimum rim width (BMO-MRW) has more recently gained prominence as an equally important biomarker for detection of glaucoma and its monitoring.^4^^,^^5^ The BMO-MRW is the minimum distance from the inner opening of the BMO to the internal limiting membrane and provides a more geometrically accurate assessment of the neuroretinal rim, or the RGC axonal complement of a given eye.^6^^,^^7^

The existing studies that have compared the 2 biomarkers in terms of their diagnostic accuracy and structure-function correlations have yielded mixed results. Some favor RNFL thickness, wheras others favor BMO-MRW; some studies reported that a combination of both parameters may be the superior choice.^8^, ^9^, ^10^ We recently showed that longitudinal RNFL measurements detected structural glaucoma changes more efficiently compared with longitudinal BMO-MRW.^11^ Discrepancies between these 2 parameters have been observed topographically at the hemidisc level, with the superior hemidisc defined as the combination of the superotemporal and superonasal sectors and the inferior hemidisc defined as the combination of the inferotemporal and inferonasal sectors on the OCT report.^12^ However, a more detailed examination of such discrepancies and their sources is lacking. This is especially relevant for monitoring glaucoma and in cases of advanced optic nerve thinning where detection of change becomes more challenging.

This cross-sectional study aims to compare the RNFL and BMO-MRW measurements when either one has fallen below the first percentile range on the temporal-superior-nasal-inferior-temporal (TSNIT) curve in a cohort of eyes with glaucoma. We sought to identify the topographic distribution of the observed discrepancies between the RNFL and BMO-MRW measurements and explore potential contributing anatomical factors.

## Methods

### Study Sample

Data from 118 patients enrolled in the Advanced Glaucoma Progression Study (AGPS), an ongoing, prospective, longitudinal study at the University of California, Los Angeles, were analyzed. Institutional Review Board approval was obtained for this study. The study adhered to the tenets of the Declaration of Helsinki and conformed to the Health Insurance Portability and Accountability Act policies. All patients provided written informed consent at the time of enrollment in the study. Each patient had an index eye enrolled in AGPS. The index eye had either moderate to advanced glaucoma or evidence of central damage and met the following inclusion criteria: a) clinical diagnosis of primary open-angle glaucoma, pseudoexfoliative glaucoma, pigmentary glaucoma, or primary angle-closure glaucoma; b) visual field mean deviation (MD) of −6 dB or worse or evidence of central damage on 24-2 visual field, defined as ≥2 points within the central 10° with *P* < 0.05 on the pattern deviation plot. The nonindex eye from the same patient, which was not included in AGPS, was also included in this study if glaucomatous damage was present. These nonindex eyes encompassed all stages of disease severity and were included to increase the power of the study because glaucoma damage is frequently localized and advanced RNFL or neuroretinal rim thinning can occur at any stage of the disease. A total of 186 index and nonindex glaucoma eyes with early to advanced disease was evaluated in this study. The following were exclusion criteria: baseline age <40 years or >80 years, best-corrected visual acuity worse than 20/50, refractive error exceeding 8 diopters of sphere or 3 diopters of cylinder, and any significant retinal or neurological disease potentially affecting OCT measurements. Study eyes had no other ocular pathology at baseline and underwent clinical exams, imaging, and visual field testing approximately every 6 months. All eyes had at least 18 months of follow-up and at least 3 OCT imaging sessions. Measurements from each patient’s final available visit were used for this cross-sectional study so that the structural biomarkers were most likely to include areas of severe damage.

### Optic Nerve Head OCT

The Spectralis spectral-domain OCT (Heidelberg Engineering) was used to obtain ONH volume scans with the Glaucoma Module Premium Edition software. The RNFL thickness measurements were acquired with a 12° measurement circle (3.5 mm in an emmetropic eye). The measurement circle is centered on the BMO centroid and consists of 768 individual A-scans along the circle. The ONH scan also acquires 24 radial B-scans centered on the BMO centroid to estimate BMO-MRW thickness. The RNFL segmentation and detection of BMO endpoints for BMO-MRW thickness are performed automatically by the Glaucoma Module Premium Edition software (Heidelberg Engineering). Images were reviewed for centration, segmentation errors, and image artifacts. Any obvious segmentation errors were manually corrected with the spectral-domain OCT device's built-in software. If any volume scans were of inadequate quality or showed poor segmentation that could not be rectified, both RNFL and BMO-MRW data for that session were excluded from the analyses. A low-quality OCT image was defined based on a quality factor <15, >10% missing data or inadequate segmentation, or any artifacts. After segmentation, the RNFL and BMO-MRW thickness measurements were exported to a personal computer.

### Mismatch Analyses

**Figure 1:**
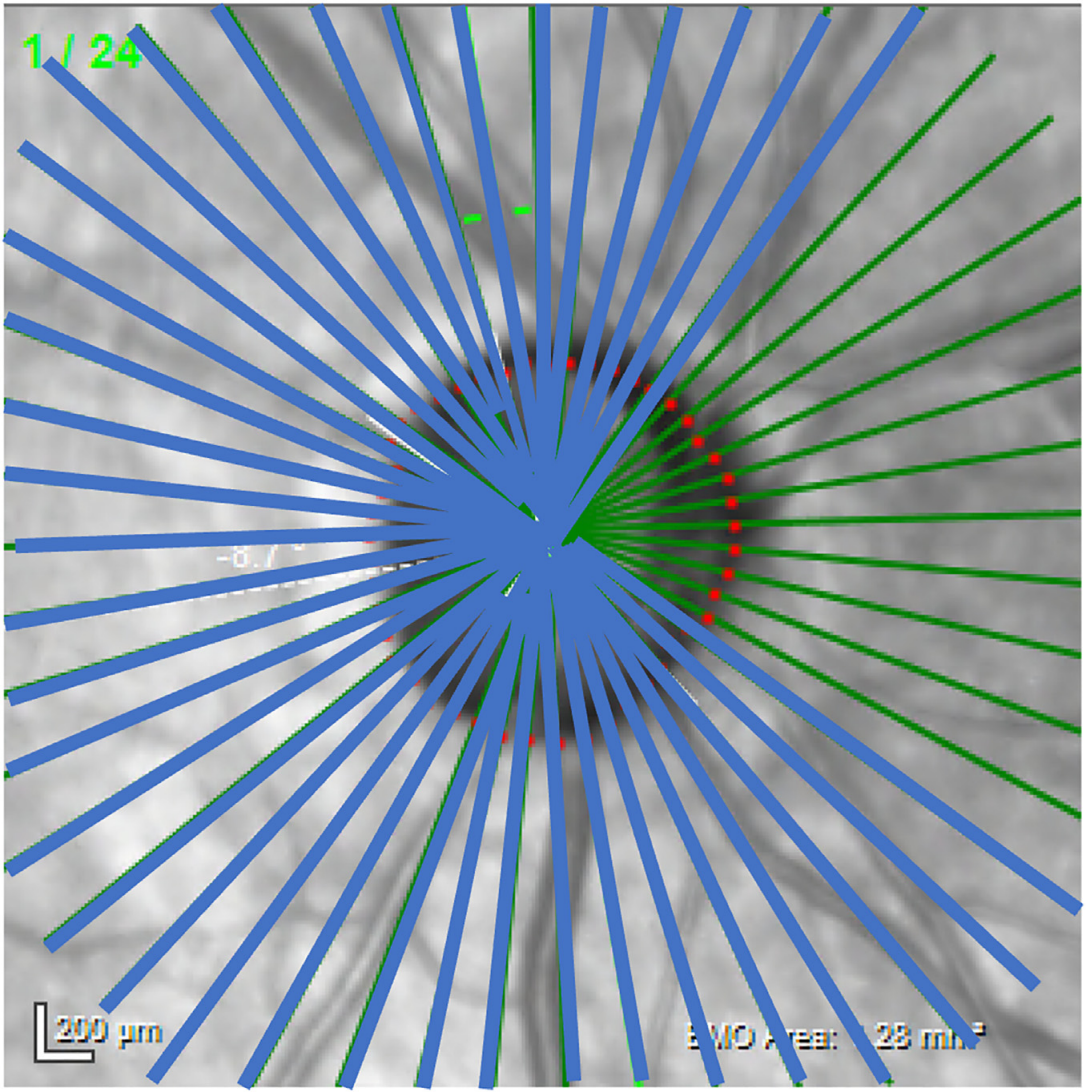
Sectoral cuts of the Bruch’s membrane opening-minimum rim width measurements. The temporal 180°, superonasal 45°, and inferonasal 45° (37 cuts spaced every 7.5° from 225° to 135°) are highlighted by the blue lines in this right eye.

The RNFL and BMO-MRW measurements from each patient’s final visit were used for this study. The 24 radial line scans of the BMO-MRW result in 48 individual thickness measurements (sectors) at 7.5° increments around the ONH. The 768 individual RNFL measurements were averaged into 48 corresponding sectors, with each sector thickness estimated as the average of 16 measurements centered around the radial BMO-MRW measurements spaced every 7.5°. Measurements from 225° to 135° centered on the BMO centroid (37 sectors) were used for mismatch analysis. This encompasses the temporal 180°, and the superonasal and inferonasal 45° of the optic nerve. The nasal 90° was excluded because it contains a significant amount of noise on the BMO-MRW measurements and is less clinically relevant. Figure 1 demonstrates the topographic distribution of the included linear B-scans around the ONH. The sectoral BMO-MRW and RNFL measurements within each eye were compared against each other to look for mismatching sectors.

An initial screen was performed to identify mismatched data points (i.e., sectors where the RNFL was high, but the BMO-MRW was low and vice versa). The data were sorted into 4 groups as follows: group 1 (RNFL ≥65 μm; BMO-MRW ≥130 μm), group 2 (RNFL ≤50 μm; BMO-MRW ≥130 μm), group 3 (RNFL ≤50 μm; BMO-MRW ≤100 μm), group 4 (RNFL ≥65 μm; BMO-MRW ≤100 μm). These numeric cutoffs from the initial screen yielded a frequency ratio of 0.73 for the number of points with BMO-MRW ≤100 μm versus the number of points with RNFL ≤50 μm. This suggests a relatively equal specificity between the 2 for this dataset, which makes these cutoffs an acceptable approximation for the first step of the screening process. Groups 2 (high BMO-MRW; low RNFL) and 4 (high RNFL; low BMO-MRW) contained possible mismatching sectors and were subjected to a secondary screen.

**Figure 2:**
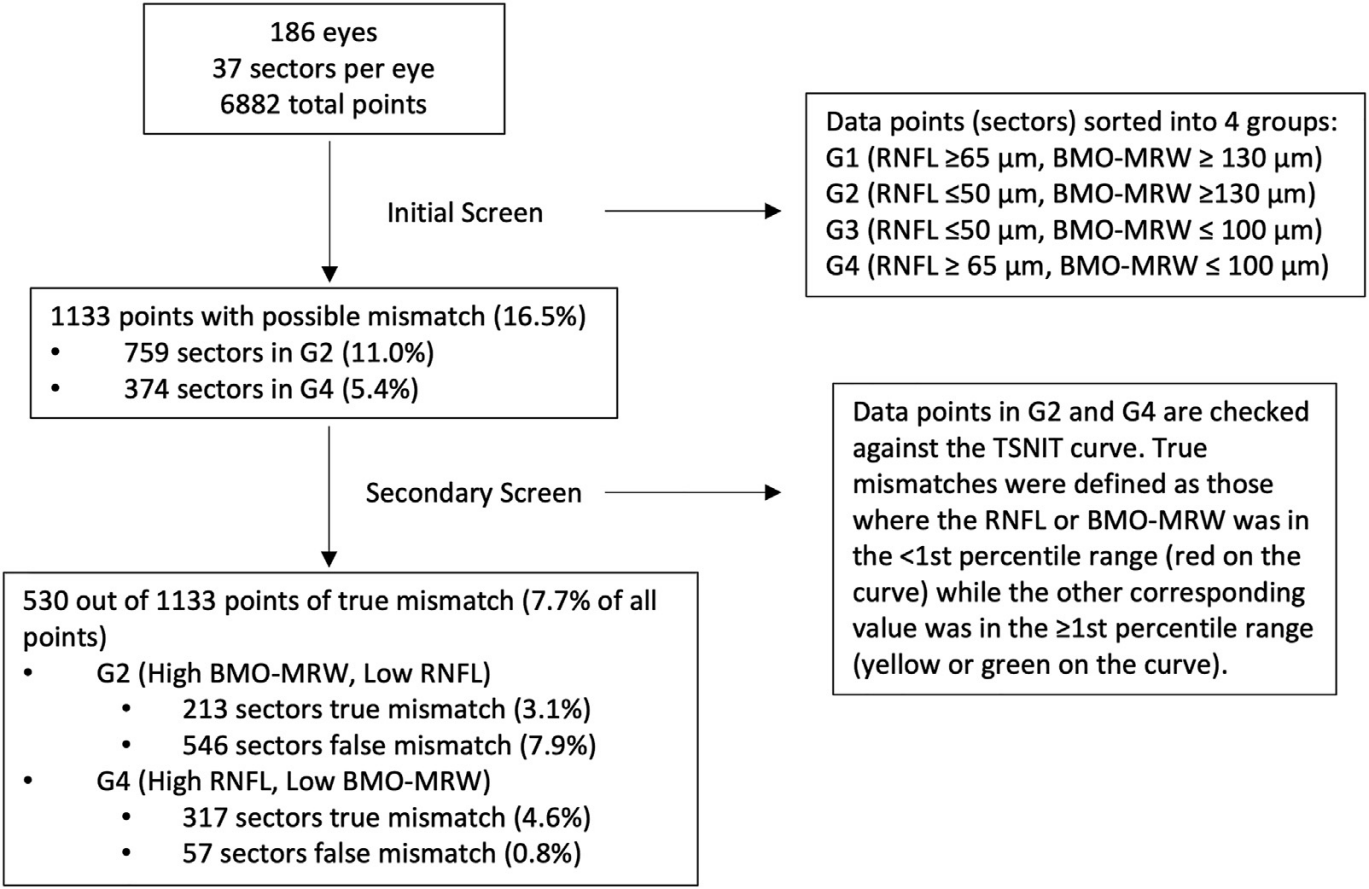
Flow chart for data screening to identify sectors where there is true mismatch between corresponding retinal nerve fiber layer thickness and Bruch’s membrane opening-minimum rim width. BMO-MRW = Bruch membrane opening-minimum rim width; RNFL = retinal nerve fiber layer; TSNIT, temporal-superior-nasal-inferior-temporal.

The secondary screen defined true mismatches as sectors where either the RNFL or BMO-MRW measurement was in the less than first percentile range (red on the TSNIT curve), whereas the other corresponding measurement was in the greater than first percentile range (yellow or green on the TSNIT curve). The RNFL and BMO-MRW measurements in each sector in groups 2 and 4 were reexamined to identify the true mismatches. We also qualitatively reviewed the cross-sectional raw OCT images at mismatching sectors to identify and categorize reasons for the mismatch. Figure 2 summarizes the entire screening process.

## Results

Table 1 describes clinical and demographic characteristics of the study patients. One hundred eighty-six eyes from 118 patients with glaucoma were included in the final analysis. The mean (standard deviation, [SD]) age of the patients was 68.1 (SD: 9.1) years at the final visit. The mean global RNFL and BMO-MRW thickness was 62.4 (SD: 14.7) and 161.9 (SD: 57.7) μm, respectively. The mean 24-2 visual field MD at baseline was −7.0 (SD: 5.9) dB. Early glaucoma was defined as MD >−6 dB, moderate glaucoma was defined as MD ≤−6 dB and ≥−12 dB, and advanced glaucoma was defined as MD <−12 dB. The distribution of disease severity at baseline included 105 eyes (56%) with early glaucoma, 42 eyes (23%) with moderate glaucoma, and 39 eyes (21%) with advanced glaucoma. Of the 105 eyes with early glaucoma, 65 eyes (35% of total) had central damage.

**Table 1:** Demographic Characteristics of the Study Eyes

No. of Eyes (Patients)	186 (118)
Age (mean ± SD, yrs)	68.1 ± 9.1
Female, n (%)	116 (62%)
Ethnicity, n (%)	
White	79 (42%)
Asian	38 (20%)
Black or African American	20 (11%)
Hispanic or Latino	17 (9%)
American Indian	2 (1%)
N/A	30 (16%)
Stage of disease at baseline, n (%)	
Early	105 (56%)
With central damage	65 (35%)
Without central damage	40 (21%)
Moderate	42 (23%)
Advanced	39 (21%)
24-2 visual field mean deviation at baseline (mean ± SD, dB)	−7.0 ± 5.9
Axial length at baseline (mean ± SD, mm)	24.6 ± 1.4
Bruch’s membrane opening (BMO) area at final visit (mean ± SD, mm^2^)	1.92 ± 0.48
Global BMO-minimum rim width at final visit (mean ± SD, μm)	161.9 ± 57.7
Global retinal nerve fiber layer thickness at final visit (mean ± SD, μm)	62.4 ± 14.7

dB = decibels; SD = standard deviation.

### Distribution of RNFL and BMO-MRW Mismatches

**Figure 3:**
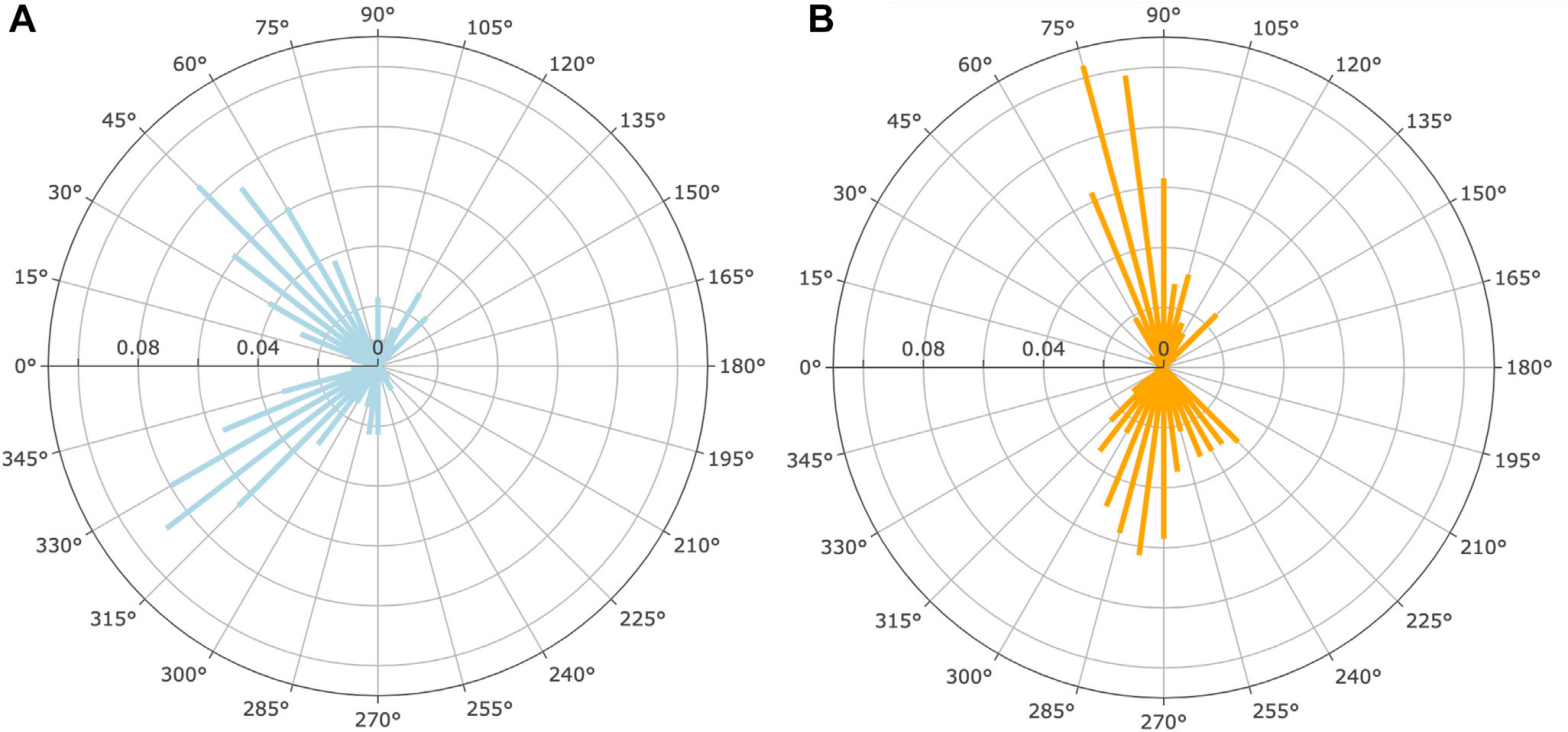
Polar charts representing the spatial distribution of mismatched sectors along the temporal 180°, superonasal 45°, and inferonasal 45° of the optic nerve head in cases of **A,** high Bruch’s membrane opening-minimum rim width and low retinal nerve fiber layer thickness and **B,** high retinal nerve fiber layer and low Bruch’s membrane opening-minimum rim width thickness. Data are presented in right eye format.

After conducting the initial and secondary screening procedures, 530 of 6882 sectors (7.7%) were identified as true mismatches, as shown in Figure 2. Of these 530 sectors, 213 sectors (3.1% of total) demonstrated a high BMO-MRW with low RNFL thickness, and 317 sectors (4.6% of total) demonstrated a high RNFL with low BMO-MRW thickness. Of these 530 sectors, 109 sectors (21%) included high BMO-MRW with low RNFL and high RNFL with low BMO-MRW sectors that occurred in the same eye. However, these 2 groups never occurred in adjacent sectors and were separated by at least 15° in the same eye. Figure 3 displays the spatial distribution of these mismatches along the ONH in the right eye format. Mismatches with a high BMO-MRW and a low RNFL were most frequently located within a 15° area centered at the 45° and 322.5° axes on the ONH, with the peak occurrence (9% of these mismatches) at 322.5° (Fig 3A). Conversely, mismatches consisting of a high RNFL and a low BMO-MRW were most frequently observed along the 15° sector spanning the superior and inferior poles of the ONH, with the peak occurrence (10% of these mismatches) at 75°, as shown in Figure 3B.

### Contribution of Blood Vessels to RNFL and BMO-MRW Mismatches

Most of the mismatches identified between the RNFL and BMO-MRW measurements were attributed to the differential impact of blood vessels. Table 2 displays the extent to which blood vessels were responsible for these discrepancies. In this context, “large blood vessels” refer to the primary branches emanating from the central retinal artery or vein that constitute the core of the superior or inferior vascular arcades. In contrast, “small blood vessels” pertain to the finer circumlinear vasculature.

**Table 2:** Proportion of Mismatches between the RNFL and BMO-MRW in Corresponding Sectors Caused by Blood Vessels

	Large Blood Vessels	Small Blood Vessels	Other	Total Sectors
High BMO-MRW, Low RNFL	50 (23.5%)	84 (39.4%)	79 (37.1%)	213
High RNFL, Low BMO-MRW	288 (90.9%)	2 (0.6%)	27 (8.5%)	317

BMO-MRW = Bruch’s membrane opening-minimum rim width; RNFL = retinal nerve fiber layer.

**Figure 4:**
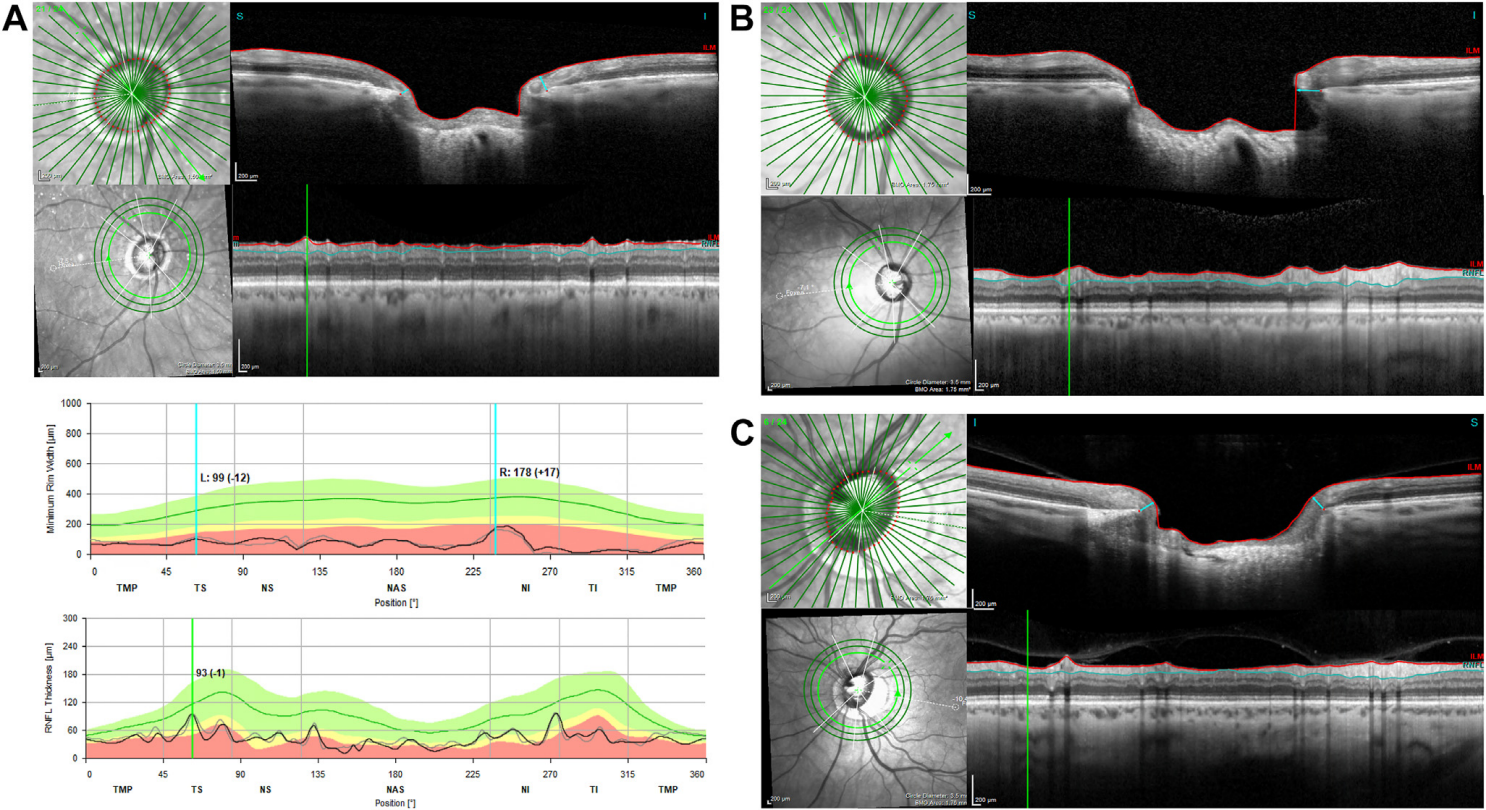
Blood vessel contributions at sectors with Bruch’s membrane opening-minimum rim width (BMO-MRW) and retinal nerve fiber layer (RNFL) mismatch. **A,** A large blood vessel thickens the RNFL more than the BMO-MRW at the 60° sector. **B,** A large blood vessel preferentially thickens the RNFL at the 75° sector due to the angular offset between the site of vessel insertion at the optic nerve head and the trajectory of the vessel in the retina. **C,** A small blood vessel preferentially thickens the BMO-MRW at the 52.5° sector. IT = inferotemporal; NAS = nasal; NS = superonasal; TMP = temporal; TS = superotemporal.

Large blood vessels accounted for the majority (90.9%) of the mismatches with high RNFL and low BMO-MRW. This discrepancy was predominantly because of a more pronounced thickening of the RNFL compared with the BMO-MRW, as depicted in Figure 4A. Furthermore, the angular distance between the point of insertion of the large blood vessels at the optic disc and their trajectory within the retinal tissue contributed to a relative thickening of the RNFL compared with the BMO-MRW, as depicted in Figure 4B. Depending on the anatomy of the vessels and eye laterality, such angular deviation also caused a relative thickening of the BMO-MRW compared with the RNFL in some cases and contributed to a smaller proportion (23.5%) of mismatches characterized by high BMO-MRW and low RNFL values. Conversely, the largest proportion (39.4%) of mismatches displaying high BMO-MRW and low RNFL thickness values was due to the presence of small blood vessels; nonvascular factors accounted for 37.1% of such mismatches (see below). The small vessels were particularly implicated in instances in which a thicker BMO-MRW occurred compared with RNFL, as depicted in Figure 4C.

### Other Causes of RNFL and BMO-MRW Mismatches

**Figure 5:**
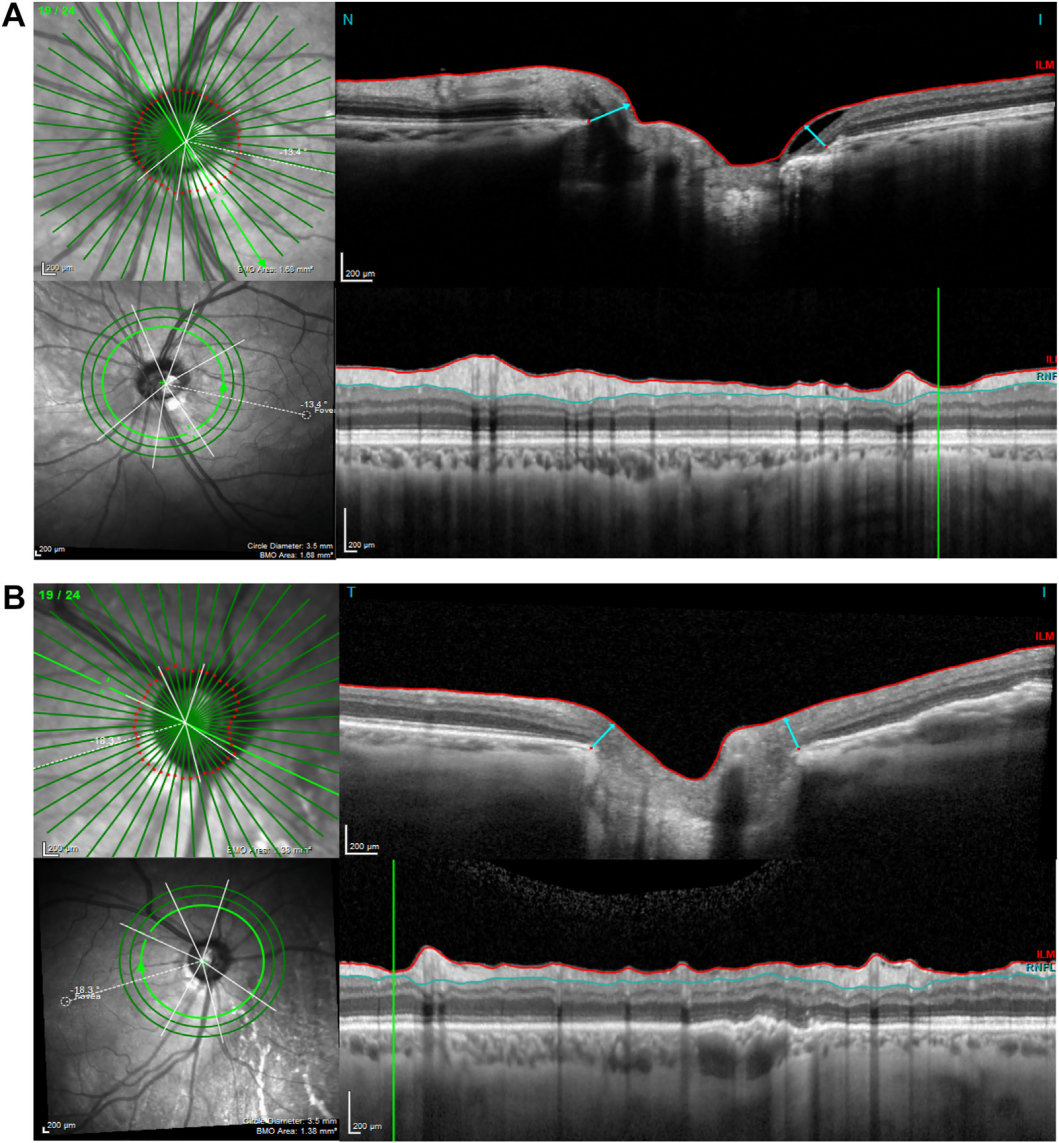
Other causes of preferential Bruch’s membrane opening-minimum rim width (BMO-MRW) thickening over the retinal nerve fiber layer. **A,** A focal area of retinoschisis preferentially thickens the BMO-MRW at the 315° sector. **B,** Inclusion of outer retinal layers contributes to preferential BMO-MRW thickening at the 45° sector.

The RNFL and BMO-MRW thickness mismatches not attributable to blood vessels were primarily seen in the high BMO-MRW and low RNFL group as shown in Table 2. Within this group, 6 of 79 mismatching sectors (7.6%) were due to focal areas of retinoschisis, resulting in BMO-MRW thickening without concomitant RNFL thickening, as shown in Figure 5A. Inclusion of the outer retinal layers such as the outer nuclear and plexiform layers in the BMO-MRW measurements, as shown in Figure 5B, was also another contributing factor. The magnitude of this inclusion of outer retinal layers was difficult to quantify.

## Discussion

Our study focused on a detailed topographic evaluation of the discrepancies between 2 structural biomarkers for glaucoma, BMO-MRW and RNFL thickness, in areas of advanced thinning on the optic nerve. The temporal 180°, superonasal 45°, and inferonasal 45° of the ONH were included for analysis. The nasal 90° was excluded because it has an abundance of BMO-MRW artifacts because of crowding of blood vessels and is less clinically relevant. Our findings revealed an overall low rate of BMO-MRW and RNFL thickness mismatches (7.7%); these mismatches were relatively evenly divided between the high BMO-MRW vs. low RNFL and high RNFL vs. low BMO-MRW groups. However, the distribution of these 2 types of mismatches around the ONH differed significantly with distinct contributing factors for each group.

Retinal blood vessels accounted for the majority (80%) of the observed mismatches. Specifically, the major branches of the central retinal artery and vein were responsible for nearly all the high RNFL vs. low BMO-MRW mismatches (90.9%). This pattern also explained why these mismatches were predominantly located along the superior and inferior poles of the ONH. These larger vessels typically lead to a more extensive thickening of the RNFL relative to the BMO-MRW as they are largest near the ONH poles and the RNFL bundles tend to follow their course. In fact, Hood et al have shown that roughly 13% of the total circumpapillary RNFL thickness is accounted for by blood vessels.^13^ These larger vessels have also been shown to contribute to a higher proportion of RNFL segmentation errors in the superior and inferior sectors as compared with the horizontal sectors, which have been accounted for in our study.^14^, ^15^, ^16^

Conversely, the high BMO-MRW with low RNFL group had a smaller contribution from blood vessels (62.9%), with a more significant influence originating from small circumlinear vessels. These vessels led to an increase in the BMO-MRW thickness without a corresponding thickening of the RNFL; hence, such mismatches were most prominent at the 45° and 322.5° sectors on the ONH. However, a sizable portion (37.1%) of the mismatches in this group was not related to blood vessels. Focal retinoschisis and inclusion of the outer retinal layers in the BMO-MRW measurements played a role in some eyes, but no other clear anatomical factors were identified. We originally hypothesized that a smaller ONH might contribute to this phenomenon, because BMO-MRW thinning might not be adequately detected because of tissue crowding and the smaller circumference of the ONH.^17^^,^^18^ However, this phenomenon was observed with similar frequency in optic discs of all sizes, thus disproving this factor as a major cause of RNFL vs. BMO-MRW thickness discrepancy.

Additionally, our study suggests that there are differences between the potential measurement floors of the RNFL and BMO-MRW. The analysis was performed on discrepancies between the RNFL and BMO-MRW in which one value was in the below the first percentile range on the TSNIT curve whereas the other was not. Therefore, sectors in which both values were already below the first percentile cutoff (i.e., potentially at the measurement floor) were excluded. Meanwhile, sectors win which one value was potentially near or at the measurement floor, while the other was not, were included. These sectors were, in fact, the ones worth highlighting, because using both structural measurements could provide a better overall picture of progression compared with either measurement alone in these cases.

These results underscore some of the limitations of current OCT technology in assessing RNFL and BMO-MRW thickness. Although previous studies have demonstrated that large blood vessels can contribute to RNFL thickening,^13^^,^^19^ our study highlights their significant impact in eyes with more advanced glaucoma damage, particularly evident in eyes with high RNFL vs. low BMO-MRW mismatches. Additionally, this study introduces a novel observation: the role played by small vessels within the ONH leading to BMO-MRW thickening. These findings suggest the potential advantages of using both parameters in conjunction, allowing one to compensate for the deficiencies of the other. Furthermore, the angular discrepancy between the vessel insertion point at the ONH and its actual trajectory in the retina can lead to a corresponding offset in BMO-MRW and RNFL thickening. Unfortunately, current technology does not fully account for this factor. Therefore, integrating RNFL and BMO-MRW measurements in clinical practice offers a synergistic approach to glaucoma detection and management. The combined structural information from these techniques provides a more comprehensive assessment of glaucomatous damage, enabling earlier diagnosis and more precise monitoring of disease progression, especially in advanced stages. Further advancements in the OCT technology could be useful in eliminating the effect of large blood vessels on the RNFL measurements and small blood vessels on the BMO-MRW measurements, and future studies could evaluate cross-sectional and longitudinal associations between these 2 structural biomarkers more effectively once these advancements are made.

The main limitation of the current study is that it may not have included all the mismatched data points. This limitation arose because the initial screening process employed empirical numeric cutoffs for RNFL and BMO-MRW thickness; the TSNIT percentile range for these parameters varies depending on factors such as patient age and the location around the ONH. For example, an RNFL measurement of 70 μm may fall below the first percentile range (red on TSNIT curve) for the superotemporal or inferotemporal cuts but may fall within the normal (>5%) range (green on TSNIT curve) for the temporal cuts. Readily exportable TSNIT percentile data for the cross-sectional BMO-MRW measurements are not available. Consequently, the double screening method was chosen as the most suitable approach to ensure specificity while still gathering sufficient data to detect underlying anatomical patterns. The other limitation is due to the fact that the BMO-MRW is measured at 7.5° intervals, whereas the RNFL thickness is measured at a much higher resolution (768 pixels) by the Spectralis OCT device, resulting in a mismatch in the resolution of the 2 biomarkers.

In conclusion, our study highlights the topographic discrepancies between circumpapillary RNFL and BMO-MRW measurements in eyes with established glaucoma, in which many areas of the ONH demonstrate significant thinning or loss of RGC axons. Although overall agreement between these biomarkers is good in areas of advanced damage, our findings emphasize the significant impact of blood vessels, both large and small, on these discrepancies. Large vessels tend to lead to RNFL thickening relative to BMO-MRW, whereas small vessels contribute to BMO-MRW thickening without a corresponding RNFL increase. Additionally, focal retinoschisis and inclusion of outer retinal layers in BMO-MRW measurements were identified as contributing factors. These findings underscore the limitations of current OCT technology and suggest the potential benefits of combining both parameters to improve glaucoma monitoring.
